# Humans Outperform Multimodal Large Language Models (MLLMs) in Non-Verbal Tests of Mental Rotation and Figural Reasoning

**DOI:** 10.3390/jintelligence14090219

**Published:** 2026-09-11

**Authors:** Jonas Lesigang, Jakob Pietschnig

**Affiliations:** 1 Department of Developmental and Educational Psychology, Faculty of Psychology, University of Vienna, 1010 Vienna, Austria; jakob.pietschnig@univie.ac.at; 2 Vienna Doctoral School in Cognition, Behavior, and Neuroscience (VDS CoBeNe), University of Vienna, 1010 Vienna, Austria

**Keywords:** artificial intelligence, human intelligence, ChatGPT, Gemini, mental rotation, figural reasoning, progressive matrices

## Abstract

Multimodal large language models (MLLMs) have become increasingly proficient at solving complex problems. Here, we systematically compare performance between humans and two of the currently most widely used MLLMs, ChatGPT-5 and Gemini 3, on mental rotation and figural reasoning tests using identical instructions for humans and online interface-instructed MLLMs. Furthermore, we aimed to assess the effect of prompting configurations (self-consistency, segmented uploads, different contexts, explicit Chain-of-Thought prompting, German vs. English prompts) on MLLM performance. To this end, we assessed a human online sample (total *N* = 430) as well as ChatGPT-5 and Gemini 3 performance on five mental rotation and figural reasoning tests in a cross-sectional comparative design. MLLM performance was evaluated via percentile ranks. The human online sample outperformed MLLMs in both mental rotation (MLLM percentile rank ranges 0 to 7 on most tests and conditions) as well as figural reasoning (MLLM percentile ranks ranges 0 to 79) tests. Prompting configuration changes increased ChatGPT-5 but not unequivocally Gemini 3 performance (mean score change ranges: 0.67 to 2.33 and −1.67 to 1.50 points, respectively). Combining best-performing prompting configurations in single comparisons yielded largest increases in both models (3.83 and 2.33 points, respectively). Our results indicate that humans may currently outperform widely used MLLMs in mental rotation and figural reasoning tasks, especially if they require complex visuospatial abilities.

## 1. Introduction

Since the 2022 release of OpenAI’s ChatGPT (OpenAI, 2025a), an advanced large language model (LLM) chatbot, average weekly user numbers have surged to hundreds of millions in 2025 (Chatterji et al., 2025), and monthly users are estimated to have surpassed one billion globally (Kemp, 2025). While even early releases impressed with unprecedented capabilities such as complex creative text generation, newer versions and competitor models were substantially refined and improved. Indeed, modern cutting-edge LLMs such as ChatGPT-5 (OpenAI, 2025b) or Gemini 3 (Pichai et al., 2025) promise to excel in problem solving in various domains such as math, software coding, or multimodal perception. Such multimodal LLMs (henceforth: MLLMs) have been shown to increasingly outperform humans in certain domains (Pichai et al., 2025).

However, it seems that exceptional MLLM performance to date is limited to specific domains. For example, even comparatively early MLLMs scored in the upper normal and above average range on text-based parts of psychometric intelligence tests (King, 2023), but were outperformed by humans on math problems (Dao & Le, 2023; Frieder et al., 2023; Lu et al., 2024). Furthermore, substantial within-MLLM performance differences across different problem domains were observed (Abdelkarim et al., 2025; Hickman et al., 2024). In general, MLLMs appear to perform better in verbal domains compared to non-verbal ones like numerical or visual problem solving. For example, MLLMs performed notably better in a missing word than in a missing number task (Abdelkarim et al., 2025) and in text-based adaptations of different tasks compared to their visually based counterparts (Haznitrama et al., 2026). In fact, MLLMs have been observed to be outperformed by humans in non-verbal cognitive tasks, sometimes barely showing chance-level item solution probabilities (Cai et al., 2025; Cao et al., 2025; Stogiannidis et al., 2025).

Among the most commonly used non-verbal cognitive tests for evaluating human cognitive abilities are mental rotation and figural reasoning tasks. On the one hand, mental rotation ability refers to the ability to mentally visualize transformations of complex patterns and is frequently assessed by Shepard–Metzler-type tests (Shepard & Metzler, 1971) in which test takers must decide whether a certain stimulus figure can be rotated to match a target figure.

On the other hand, figural reasoning refers to the ability to apply inductive reasoning based on figural stimuli. Figural reasoning is frequently assessed by Raven-type progressive matrices (Raven, 1941) in which participants have to complete 3 × 3 symbol matrices by inferring and applying underlying matrix progression rules.

Whilst some previous studies observed MLLMs to perform below human averages both in mental rotation (Hayashi & Hirata, 2026; Stogiannidis et al., 2025) and figural reasoning (Cao et al., 2025; Haznitrama et al., 2026) tasks, evidence from other studies contrast these findings. Specifically, perceptual reasoning performance on vectorized images and verbally contextualized subtests of the Wechsler Adult Intelligence scale yielded above average MLLM performance compared to humans (MLLM IQs = 118–125 points; Huang & Li, 2024; Wasilewski & Jablonski, 2024). This may indicate that differences in MLLM non-verbal cognitive task performance may be attributable to differences in test instruments, non-verbal domain, user decisions preceding the MLLM responses such as prompting specifications or preprocessing steps, or a combination of these variables.

There are several further potentially meaningful influences that may affect MLLM task performance in comparison to humans. First, due to rapid technological advances in this field (Jaš & Gams, 2025), MLLM (non-verbal) cognitive test performance may have been increasing since past studies. Second, so far studies frequently compared different MLLMs or model versions, instead of systematically comparing MLLM performance with human performance (e.g., Balunović et al., 2025; White et al., 2024; Xiao et al., 2024; Xu et al., 2024; L. Zhang et al., 2026). Third, most studies tested MLLMs via Application Programming Interfaces (APIs). API calls allow explicit customization of model parameters, thus allowing for an economic assessment of large numbers of strictly specified trial runs. However, typical users rarely use APIs in everyday settings, but rather use online interfaces where MLLM parametrizations are usually opaque (Park et al., 2025) and therefore MLLM response behaviors may systematically differ from responses obtained from API calls (Lipphardt et al., 2026). This means that most MLLM performance benchmarks, while useful for assessing fine-tuned performance maxima, may not be directly interpretable in terms of their relevance for the average MLLM user.

This is, for example, especially relevant for estimating the impact of MLLM-assisted response behavior during unproctored online assessments. In this vein, MLLMs pose an increasing challenge to the validity of online assessments of human cognitive abilities (Hickman, 2025; Hickman et al., 2024). This development is important considering the increasing number of applicants using MLLMs to assist in unproctored online assessments (Westfall, 2024). To evaluate the susceptibility of such assessments to MLLM-assisted response behavior, continuous monitoring of human vs. MLLM abilities is warranted due to rapid MLLM performance improvements (Jaš & Gams, 2025). Such comparisons need to be systematic and conducted in naturalistic settings. More specifically, most prompts to assist in reasoning tasks are presumably obtained by simply copying task instructions to publicly available MLLM online interfaces. Consequently, imitating such behaviors may represent a naturalistic way to assess the impact of MLLMs on everyday contexts.

In a similar vein, prompting specifications can also notably impact MLLM reasoning performance. This is important because it has been shown that users systematically differ in their use of prompting strategies (H. Xue et al., 2026) and apply different prompting strategies with varying frequencies (Chien et al., 2026). Non-standard prompting configurations include, for example, Chain-of-Thought (CoT) prompting, which has been shown to increase MLLM performance (Wei et al., 2022). When using CoT prompting, MLLMs are explicitly instructed to apply multiple intermediary reasoning steps before responding (Wei et al., 2022). However, effectiveness of CoT prompting may vary between MLLMs and domains (Chen et al., 2023). Furthermore, self-consistency prompting has been shown to increase performance (Wang et al., 2023). Self-consistency prompting is based on MLLMs usually responding stochastically instead of deterministically, meaning that responses might differ if a prompt is repeated. Importantly, if a model performs above chance-level in a single-choice task while errors are random, the correct response option is expected to be selected more frequently than each incorrect response option. Consequently, majority voting using all responses has been observed to increase accuracy (Wang et al., 2023).

For image-based reasoning in particular, segmenting prompt images (e.g., uploading nine images instead of a 3 × 3 matrix image as a whole) has been shown to increase performance (Li et al., 2025; Y. Zhang et al., 2024), as is the case for providing example items before problems have to be solved (i.e., few-shot learning; Brown et al., 2020; Wu et al., 2024).

Here, we aimed to examine how performance of two of the currently most widely used MLLMs, ChatGPT-5 and Gemini 3 (Bailyn, 2026; Kemp, 2025), on different tests of mental rotation and figural reasoning abilities, compares with human performance. To this end, we administered five psychometric mental rotation and figural reasoning (matrix progression) measures to MLLMs and a human online sample. Furthermore, we systematically assessed effects of prompting configurations on MLLM performance. Finally, we explored potential effects of different matrix progression rules included in matrix progression test items (e.g., row-wise element addition vs. subtraction within matrices).

## 2. Materials and Methods

This cross-sectional comparative study was preregistered at https://osf.io/pxyv7 (accessed on 4 September 2026) before collecting any data, including hypotheses, study design, sampling plan, and analysis plan. Deviations from the preregistration (Willroth & Atherton, 2024) and anonymized test data of human participants, MLLM responses, and analysis script are provided at https://osf.io/f4nqb/files/osfstorage (accessed on 4 September 2026). This study was approved before collecting any data by the Institutional Review Board of the Department for Developmental and Educational Psychology at the University of Vienna (#03_2026).

### 2.1. Participants

#### 2.1.1. Human Online Sample

Participants were invited to complete up to five mental rotation or figural reasoning tests online. A total of 430 participants who completed at least one test without omitting any items were included in our analyses (58.60% women, 38.60% men, 2.79% other; age *M* = 29.20 years, *SD* = 14.06, range = 18–76 years; see Supplementary Materials at https://osf.io/f4nqb/files/osfstorage, accessed on 4 September 2026, for details). Sample characteristics are detailed in Table 1.

#### 2.1.2. MLLM Chatbots

We examined two of the currently most widely used MLLMs (Bailyn, 2026; Kemp, 2025), ChatGPT-5 (OpenAI, 2025a) and Gemini 3 (Google LLC, 2025). We accessed ChatGPT-5 via https://uai.univie.ac.at/ (accessed on 4 September 2026), a cloud-based ChatGPT-5 interface provided by the University of Vienna. This interface allows customization of model reasoning and verbosity parameters, which we set to high, expecting better performance with longer reasoning (Wei et al., 2022), and low, to obtain unambiguous responses, respectively. We accessed Gemini 3 via https://gemini.google.com/ (accessed on 4 September 2026) and selected the Thinking model to allow for extended reasoning, thus optimizing reasoning for everyday interface use and obtaining a similar specification as for ChatGPT-5.

### 2.2. Materials

**Figure 1:**
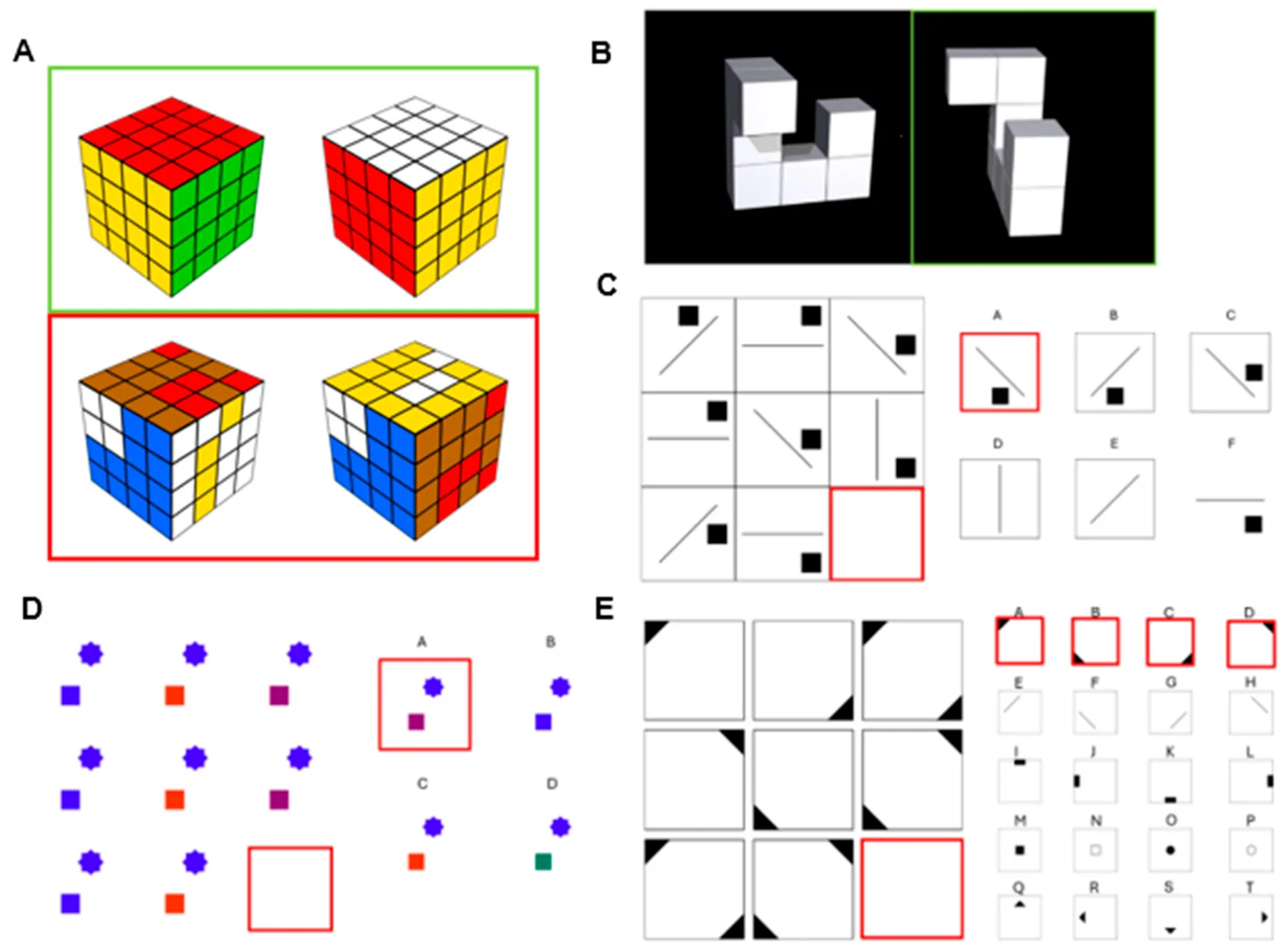
Example items of five mental rotation and figural reasoning tests administered to MLLMs and a human online sample. (**A**) Cube rotation test example items (R-Cube-SR; Fehringer, 2023). The top depicts an example item from the first test part (monochromatic cube faces), the bottom an example item from the second part (dichromatic faces). Participants must decide whether the response cube can represent a rotated perspective of the stimulus cube. (**B**) Figure rotation test example item (3D-FR; Ganis & Kievit, 2015). Participants must decide whether the response figure can represent a rotated perspective of the stimulus figure. (**C**) Black-and-white matrix progression test example item (HMT; Heydasch, 2014). Participants must decide which response option correctly completes the matrix. (**D**) Colored matrix progression test example item (MaRs; Chierchia et al., 2019). Participants must decide which response option correctly completes the matrix. (**E**) Response assembly matrix progression test example item (OMIB; Koch et al., 2022). Participants must decide which combination of response options correctly completes the matrix. All original test materials upon which these figures are based are publicly accessible under open access licenses as detailed in the Supplementary Materials at https://osf.io/f4nqb/files/osfstorage (accessed on 4 September 2026).

In this study, we used five cognitive test instruments assessing different mental rotation and figural reasoning capabilities (two mental rotation and three progressive matrices tests; example items are provided in Figure 1). The rationale for selecting these tests was fourfold. First, using multiple, comparatively recently developed tests reduced the likelihood that MLLMs were trained on (all) administered items. Second, using similar tests to assess the same cognitive domains with tests differing in terms of instruction and response formats reduced the likelihood that MLLM responses followed stereotyped solution steps based on abundant training data for one type of test (e.g., black-and-white progressive matrices with single-choice responses are commonplace and may therefore be expected to have been more frequently included in MLLM training data than other test types). Third, tests with varying item difficulty for humans (i.e., ranging from easy to hard) allowed us to assess MLLM performance across the human ability spectrum. Finally, open access items were publicly available for all selected tests, ensuring that prompting MLLMs did not breach copyright regulations.

The R-Cube-SR (Fehringer, 2023; Figure 1A) is a mental rotation test. Participants are presented with two colored 3D images of Rubik’s-type cubes with unique 4 × 4 faces and must decide whether the response cube can represent a rotated view of the stimulus cube. The test consists of two parts with 24 items each. In the first part, cube faces are monochromatic and in the second one dichromatic. The average test duration for human participants was 3 min 35 s for the first part and 4 min 43 s for the second part. Reliability estimations in two independent samples yielded McDonald’s ω = 0.62–0.71 while convergent validity estimates yielded *r* = 0.34–0.37 with another mental rotation test (Fehringer, 2023). In the present sample, participants showed a ceiling effect leading to a low Cronbach α with 0.50 in the first, and 0.57 in the second part (first part score range 18–24, second part 16–24).

For the second mental rotation test, we randomly selected 24 out of 384 available stimuli from a three-dimensional figure rotation test (3D-FR; Ganis & Kievit, 2015; Figure 1B). Participants are presented with a pair of black and white 3D images of Shepard–Metzler figures (Shepard & Metzler, 1971) with depth cues and must decide whether or not a response figure represents a rotated view of the stimulus figure. Between-item rotation angles vary from 0° to 50°, 100°, and 150°. The average test duration for human participants was 3 min 44 s. Validity was assessed by comparing test performance patterns with previous Shepard–Metzler-type tests and was deemed suitable (Ganis & Kievit, 2015). Again, Cronbach α was low in our human data with 0.62 due to ceiling effects (score range 16–24).

The HMT (Hagen Matrices Test; Heydasch, 2014; Figure 1C) is a Raven’s-type matrices test assessing figural reasoning (Raven, 1941). Participants are presented with a 3 × 3 matrix with one black-and-white symbol per cell. The bottom right cell is empty. Participants must select the missing symbol out of six options by inferring matrix progression rules which determine the progression or occurrence of symbols throughout the matrix (henceforth: black-and-white matrix progression test). The test consists of 20 items. The average test duration for human participants was 19 min 42 s. Internal consistency in prior studies was satisfactory yielding 0.78 and convergent validity was established showing appropriate correlations with general, figural, and numeric reasoning in a previous study (*r*s = 0.50–0.57; Heydasch, 2014). Cronbach α was good in our human data with 0.84.

We randomly selected 20 out of 80 available items from the MaRs (Matrix Reasoning Item Bank; Chierchia et al., 2019; Figure 1D), another Raven’s-type matrices test of figural reasoning. In this test, four response options are available per item and items are colored, introducing color-based rules (henceforth: colored matrix progression test). The average test duration for human participants was 9 min 26 s. In a previous study, split-half reliability for the 80 item version was *r* = 0.82, and convergent validity was excellent as evidenced by large correlations with another matrix reasoning test, *r* = 0.61 (Chierchia et al., 2019). Cronbach α in our human data was low with 0.54, most likely due to ceiling effects (score range 12–20).

We randomly selected one out of ten available item sets developed for covering a broad difficulty range of the OMIB (Open Matrices Item Bank; Koch et al., 2022; Figure 1E), a Raven’s-type matrices test of figural reasoning. In this test, responses are assembled by selecting the appropriate elements from 20 basic visual elements (response elements are identical across items) for each item in a multiple choice administration (henceforth: response assembly matrix progression test). The test consists of 28 items. The average test duration for human participants was 29 min 58 s. Internal consistency in a prior study was excellent, yielding Cronbach α = 0.92 (Koch et al., 2022). Cronbach α was good in our human data with 0.94.

For our exploratory investigation of the impact of different matrix completion rules on MLLM performance, the full item set of the response assembly matrix progression test (220 items, OMIB-220) was used for assessing MLLMs only. To this end, we took advantage of the systematic item construction based on different matrix progression rules in this test, where the missing symbol can be deduced by applying a combination of up to five out of six distinct matrix progression rules (addition, subtraction, disjunction, intersection, rotation, completion). Each rule was associated with additional but independent visual elements. The frequencies of specific rule combinations are approximately evenly distributed across the 220 items.

### 2.3. Procedure

#### 2.3.1. Human Online Sample

We recruited participants opportunistically between February and April 2026 via online chat groups, flyers, and word of mouth. Participation was incentivized by offering feedback on test performance. Participants had to be at least 18 years old and not suffer from color blindness to be eligible for participation.

All tests were completed via an online survey platform (https://eu.questionpro.com, accessed on 4 September 2026). After providing informed consent, participants reported their gender (female vs. male vs. diverse), date of birth, and highest educational attainment (henceforth: education). Subsequently, the tests were administered in random order for each participant. Each test was preceded by brief instructions and example items. Test items were assigned test-dependent maximum completion times (cube rotation: 30 s, figure rotation: 30 s, black-and-white matrix progression: 2 min 30 s, colored matrix progression: 1 min 30 s, response assembly matrix progression: 2 min 30 s). After completing each test, participants received feedback on the proportion of correct responses. Verbatim instructions to human participants are available in the online Supplementary Materials at https://osf.io/f4nqb/files/osfstorage (accessed on 4 September 2026).

#### 2.3.2. MLLMs

Tests for MLLMs were administered via online interfaces (https://uai.univie.ac.at/ for ChatGPT-5, https://gemini.google.com/ for Gemini 3, both accessed on 4 September 2026) between February and March 2026. When assessing MLLM performance, we started with identical introductory texts, test instructions, and example items in each administration as were used for human participants (see the Supplementary Materials at https://osf.io/f4nqb/files/osfstorage for minor differences, such as informing MLLMs at the outset “You participate in a test of visual reasoning now.”). Then, we prompted MLLMs with all items of an individual test in the same chat (i.e., in the same context window, meaning MLLMs had access to previous prompts and responses when responding to new prompts). We uploaded a single image formatted as .png per item, containing (i) both figures for mental rotation tests and (ii) the 3 × 3 input matrix as well as all response options for matrices tests.

Each image was accompanied by a brief test-dependent, German text prompt identical to the instruction text for human participants (e.g., “Which response option (A–D) is correct?”). MLLM responses were recorded. If no clear response was fed back by the MLLM or an error occurred (e.g., an empty response or an error message), the following standardized prompt was sent: “Please select a response.”. If again no equivocal response was obtained, the response was recorded as false (*n* = 35 and 0 by ChatGPT-5 and Gemini 3, respectively, across a total of 2680 scored items). Finally, to prevent potential biases based on saved chat histories, we deleted the chat history before starting each new chat (i.e., before any administration of all five test instruments). This prompting approach served as our default prompting specification.

Next, we tested the effects of different prompting configurations on MLLM mental rotation and figural reasoning performance to explore whether MLLM performance is affected by simple amendments to default prompting specifications. This approach has been successfully used before when investigating MLLMs’ visual reasoning capabilities (Cao et al., 2025; Xu et al., 2024; Y. Zhang et al., 2024). To this end, we first repeated each test ten times and scored responses by majority voting (=self-consistency configuration; partial points for ties) to explore whether this improves response accuracy (due to response heterogeneity, majority voting was performed on the level of basic visual elements for the response assembly matrix progression test).

Second, we repeated each test with segmented test items (=segmented configuration) to explore whether this improves image processing and therefore response accuracy. More specifically, instead of uploading single images, we uploaded two separate images for mental rotation tests (stimulus image and response image), and ten separate images for matrix progression tests (nine images representing the 3 × 3 matrix cells, one image representing all response options; due to repeated errors, chats had to be restarted after every fourth item in matrices tests for ChatGPT-5).

Third, we repeated each test with item-wise administration (=context configuration). Here, we started a new chat (i.e., a new context window) for each item to explore whether responses might degrade over time when having access to previous items and responses which are uninformative for novel items. In this configuration, no in-context-learning was possible except from the example items.

Fourth, we repeated each test with an appended explicit CoT prompt (=CoT configuration, “think step by step”; Chen et al., 2023) to explore whether this improves reasoning and therefore response accuracy.

Finally, we repeated each test with all instructions translated from German (i.e., our test administration language for the human sample) to English to explore whether prompting language impacts performance since MLLMs were presumably primarily trained on English content (=English configuration).

**Figure 2:**
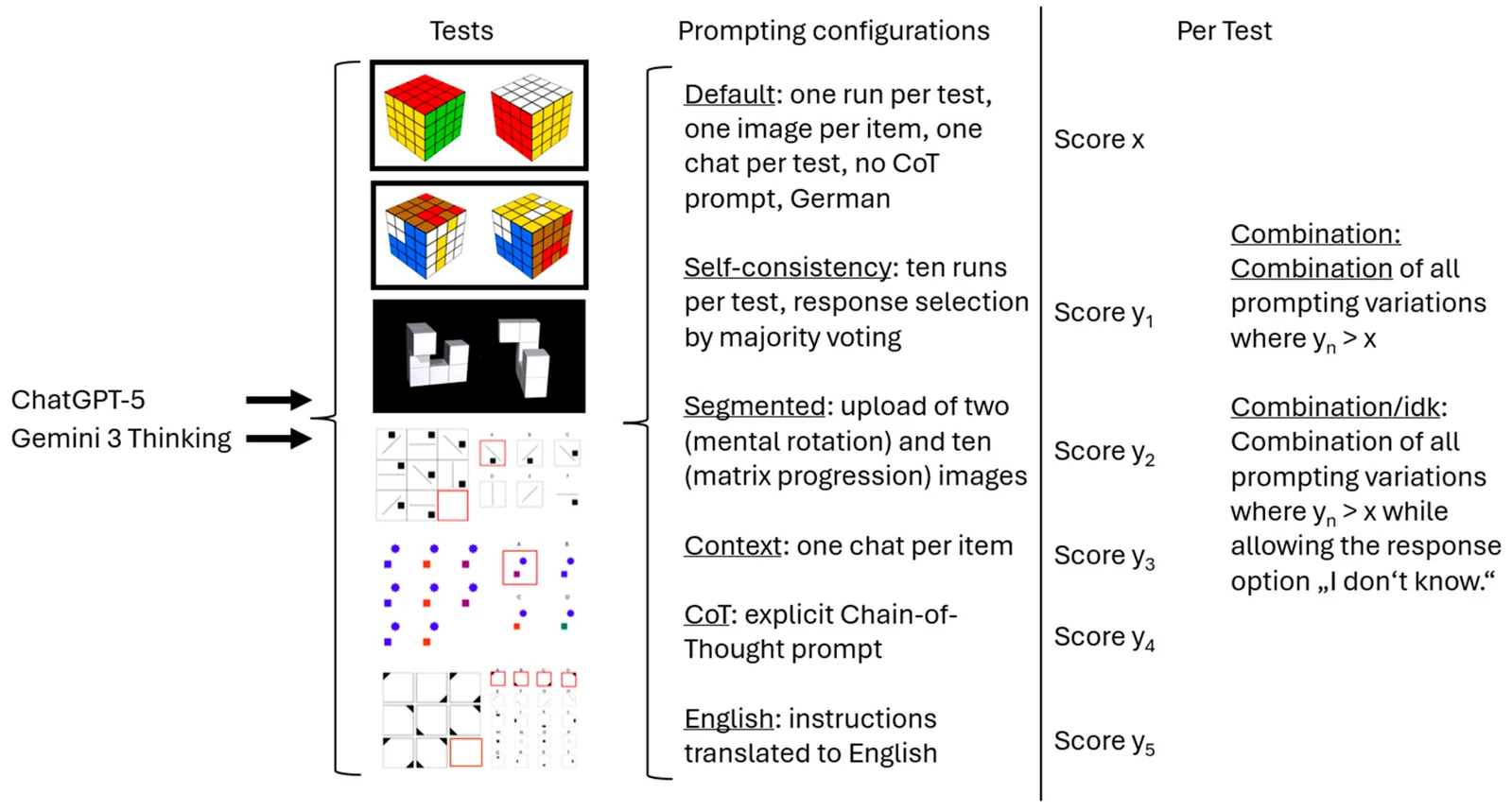
Study flow of MLLM testing. Both MLLMs were administered all tests, and each test was administered once for each prompting configuration (excepting self-consistency, where tests were administered ten times). Then, test scores per MLLM and test were compared between each prompting configuration and default prompting. All prompting configurations which outperformed default prompting were combined for another test administration. This step was repeated for an administration allowing the MLLMs to respond with “I don’t know.”. All original test materials upon which the figures are based are publicly accessible under open access licenses as detailed in the Supplementary Materials at https://osf.io/f4nqb/files/osfstorage (accessed on 4 September 2026).

Subsequently, we repeated this administration once again by using the combination of prompting configurations (=combination configuration) that yielded the best individual results per test (defined as all prompting configurations where the test score was higher than the test score obtained by default prompting specifications). We excluded the self-consistency configuration from this step due to resource constraints (i.e., depending on the other prompting configurations, a single self-consistency run might have required more than 1000 manual prompts). Next, this step was repeated while including a response option which allowed the model to specify that it did not know the correct answer to explore whether this would reduce the proportion of incorrect responses for each test (=combination/idk configuration; “You may also respond with “I don’t know.” “I don’t know” is better than being wrong, but worse than being correct.”). We administered each test to each MLLM once for each prompting configuration, excepting self-consistency, where we administered each test ten times. The MLLM study flow is summarized in Figure 2.

Finally, we tested both MLLMs on the full OMIB-220, again using the best combination of prompting configurations excepting self-consistency to explore the impact of different matrix progression rules on performance.

In all, 2680 out of 5096 item prompt responses were scored. Verbatim instructions and prompting configurations to MLLMs are reported in the Supplementary Materials at https://osf.io/f4nqb/files/osfstorage (accessed on 4 September 2026).

### 2.4. Data Analysis

All analyses were performed in R v. 4.4.2 (R Core Team, 2024) using RStudio v. 2025.09.2 (Posit Team, 2025). First, we excluded test-specific human data for performances 3 *SD*s below the mean (range *n* = 0 to 7 participants excluded across tests) because these were deemed unlikely to represent instruction-compliant performance (see Supplementary Materials at https://osf.io/f4nqb/files/osfstorage, accessed on 4 September 2026, for details).

Then, we descriptively compared MLLM performance with human performance by calculating MLLM percentile ranks per test and prompting specification. For evaluating the effect of systematic prompting configurations, we calculated the mean performance change in raw test scores across all tests when using a certain configuration compared to default prompting per MLLM.

Finally, we calculated a linear regression model for each MLLM, predicting OMIB-220 performance by all six matrix progression rules as binary variables (each rule was either required to solve an item or not) to assess if matrix progression rules had differential effects on performance.

## 3. Results

In mental rotation tests, MLLMs were almost invariably outperformed by our human online sample (Table 2). Specifically, ChatGPT-5 was outperformed by our human online sample in the first part of the cube rotation test (monochromatic faces; mean percentile rank across default prompting and all prompting configurations = 0.5, range: 0.0 to 4.0), the second part of the cube rotation test (dichromatic faces; mean percentile rank = 0.0, upper range limit < 0.01), and the figure rotation test (mean percentile rank = 1.0, range: 0.0 to 7.0). Gemini 3 was also outperformed by our human online sample in the second part of the cube rotation test (mean percentile rank = 0.9, range: 0.0 to 7.0) and figure rotation test (mean percentile rank = 0.8, range: 0.0 to 3.0) but showed a ceiling effect in the first part of the cube rotation test, akin to the human sample (mean percentile rank = 80.8, range: 11.0 to 100.0; mean human vs. Gemini 3 raw score = 23.15 vs. 23.63, respectively).

In figural reasoning, MLLMs performed, in general, better than in mental rotation tests but were still more often than not outperformed by our human online sample (Table 2). ChatGPT-5 varied considerably in performance on the black-and-white matrix progression test, yielding percentiles from chance-level to about average human performance (mean percentile rank = 18.3, range: 1.0 to 55.0) but showed substantially lower variation at the bottom of the human performance distributions in the colored matrix progression test (mean percentile rank = 4.5, range: 0.0 to 27.0) and the response assembly matrix progression test (mean percentile rank = 10.4, range: 10.0 to 11.0).

Gemini 3 achieved low average to average performance compared to our human online sample in the black-and-white matrix progression test (mean percentile rank = 45.8, range: 19.0 to 79.0) and the colored matrix progression test (mean percentile rank = 26.0, range: 0.0 to 47.0) but was clearly outperformed by human participants in the response assembly matrix progression test (mean percentile rank = 10.5, range: 10.0 to 11.0).

### 3.1. Effects of Prompting Configurations

For ChatGPT-5, any prompting configurations other than default prompting yielded better results across tests (mean raw score gains: self-consistency 0.83; segmented 0.83; context 2.33; CoT 1.00; English 0.67). However, for Gemini 3 prompting performance only improved in three out of five configurations compared to default prompting (mean raw score changes: self-consistency 1.00; segmented −1.67; context 0.67; CoT 1.50; English −0.67).

**Figure 3:**
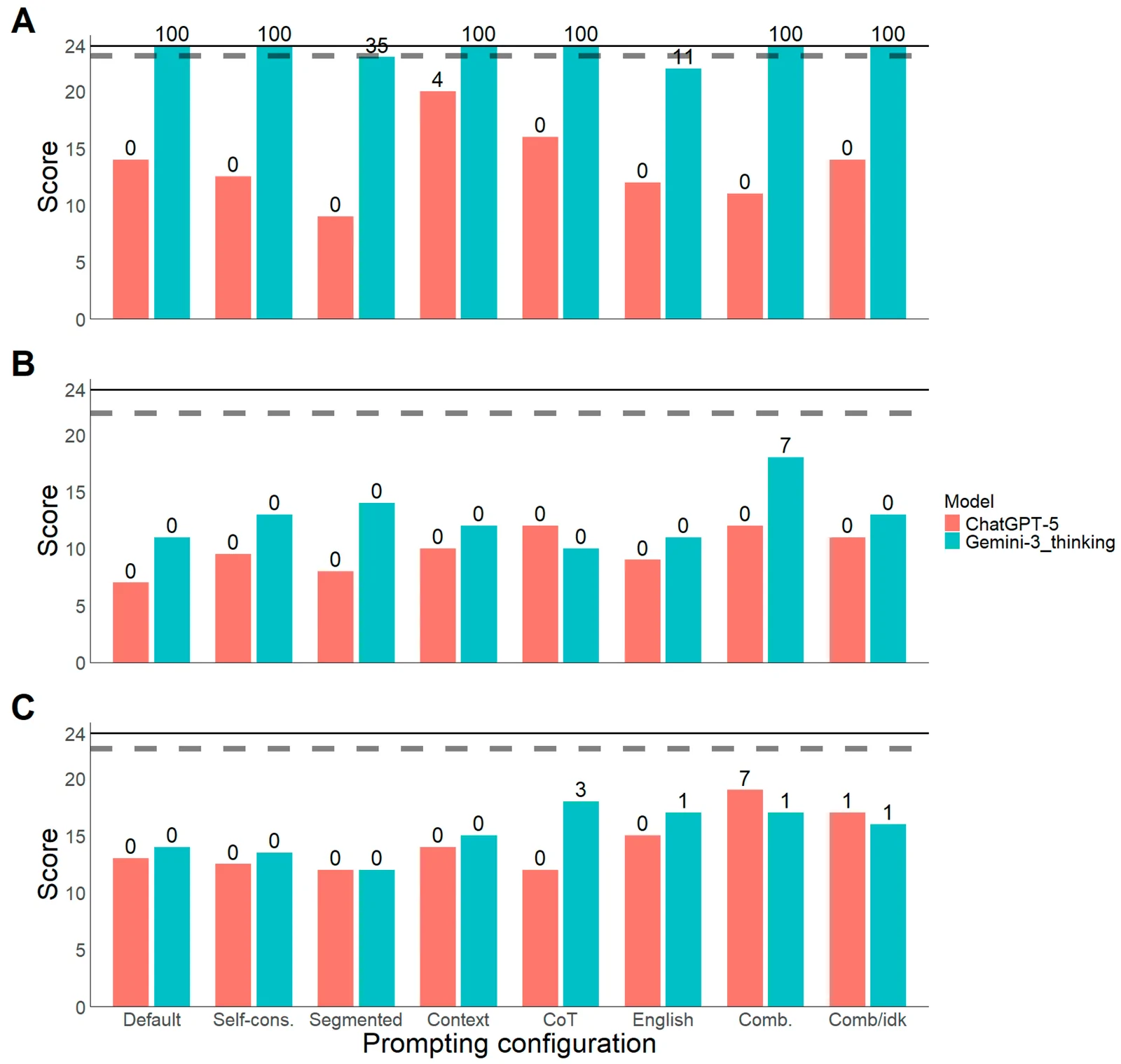
Results of comparing performance of our human online sample with MLLM performance on mental rotation tests. Bars indicate MLLM scores on (**A**) the first part of the cube rotation test (only monochromatic faces), (**B**) the second part of the cube rotation test (dichromatic faces), and (**C**) the figure rotation test. Above each bar, the percentile rank compared to our human sample is displayed. The dashed horizontal line indicates mean human performance while the solid horizontal line indicates the maximum possible test score. Default prompting included assessing MLLMs in the same chat window using single image stimuli and German instructions. In the self-consistency configuration, MLLMs were assessed ten times per test and the most frequent response per item was scored as response. In the segmented configuration, prompting stimuli did not comprise single images but were segmented into separate images (one for the stimulus and one for the response cube or figure, respectively). In the context configuration, each item was administered in a new chat (context window). In the CoT configuration, an explicit Chain-of-Thought prompt was appended to each item’s instruction (“think step by step”). In the English configuration, all instructions were translated to English. In the combination of prompting configurations, all configurations were combined which had exhibited increased performance compared to default prompting (excepting self-consistency). In the combination/idk configuration, this step was repeated while additionally allowing the model to respond with “I don’t know”.

**Figure 4:**
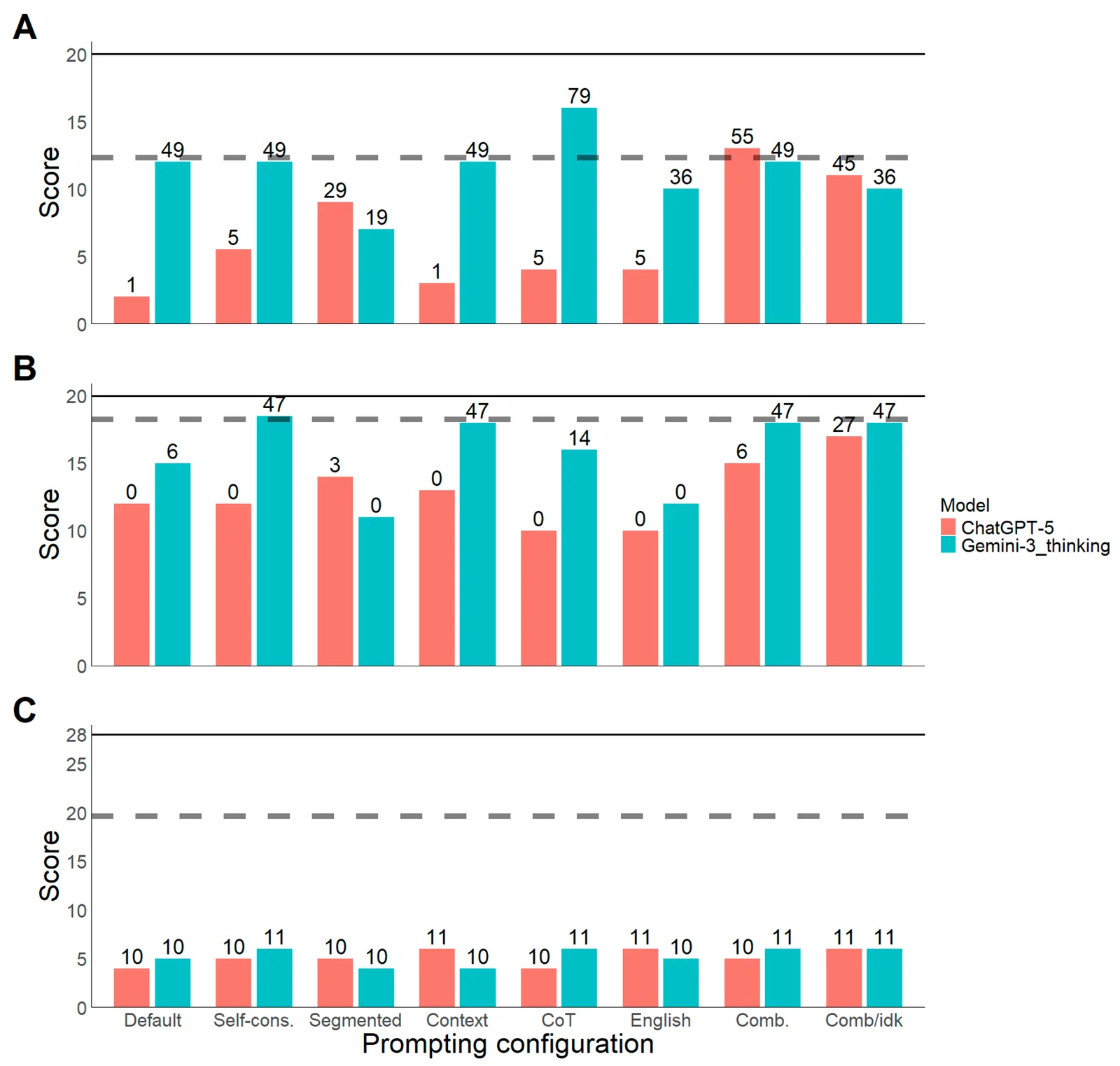
Results of comparing performance of our human online sample with MLLM performance on figural reasoning tests. Bars indicate MLLM scores on (**A**) the black-and-white matrix progression test, (**B**) the colored matrix progression test, and (**C**) the response assembly matrix progression test. Above each bar, the percentile rank compared to our human sample is displayed. The dashed horizontal line indicates mean human performance while the solid horizontal line indicates the maximum possible test score. Default prompting included assessing MLLMs in the same chat window using single image stimuli and German instructions. In the self-consistency configuration, MLLMs were assessed ten times per test and the most frequent response per item was scored as response. In the segmented configuration, prompting stimuli did not comprise single images but were segmented into separate images (nine for each cell of the 3 × 3 matrix + one for the response options). In the context configuration, each item was administered in a new chat (context window). In the CoT configuration, an explicit Chain-of-Thought prompt was appended to each item’s instruction (“think step by step”). In the English configuration, all instructions were translated to English. In the combination of prompting configurations, all configurations were combined which had exhibited increased performance compared to default prompting (excepting self-consistency). In the combination/idk configuration, this step was repeated while additionally allowing the model to respond with “I don’t know”.

Most importantly, however, for both MLLMs, the combination of best prompting configurations yielded the largest average performance increases compared to default prompting out of all prompting configurations. On average, raw test scores of ChatGPT-5 and Gemini 3 increased by 3.83 points (range 1–11) and 2.33 points (range 0–7), respectively. Results are detailed in Figure 3 and Figure 4 for mental rotation and figural reasoning tests, respectively. Additionally, boxplots with overlaid data points for individual human participants and MLLM test scores are available in the Supplementary Materials at https://osf.io/f4nqb/files/osfstorage (accessed on 4 September 2026).

The response option “I don’t know” was never used by Gemini 3, whilst ChatGPT-5 used this response option 18 out of 140 times, when available. In those two tests in which ChatGPT-5 used the “I don’t know” response option, the proportion of correct responses either remained virtually identical for dichromatic cube rotation (50% vs. 52% of correct responses for standard vs. administrations including the “I don’t know” response option) or was somewhat higher for response assembly matrix progression (18% vs. 46% correct responses for standard vs. administrations including the “I don’t know” response option).

### 3.2. Effects of Matrix Progression Rules

While the inclusion of any matrix progression rule in an item reduced the likelihood of correctly solving this specific item in the response assembly matrix progression test, we observed some differentiation in rule difficulty between MLLMs. Additionally, the completeness rule was the least detrimental matrix progression rule for both MLLMs. Finally, the total number of rules was strongly associated with performance across MLLMs (range % correct for both models for one rule: 75–85, two rules: 32–34, three rules: 4–8, four or five rules: 0; see Supplementary Materials at https://osf.io/f4nqb/files/osfstorage, accessed on 4 September 2026, for details).

## 4. Discussion

In this cross-sectional comparative study, we show that a human online sample outperformed two of the currently most widely used MLLMs, ChatGPT-5 and Gemini 3, across different mental rotation and figural reasoning tests when tested via online interfaces. MLLM performance was differentiated according to non-verbal test type with MLLMs performing comparably worse in mental rotation than in figural reasoning tests. Differences in prompting configurations systematically affected MLLM performance in our testing setup, consistently yielding largest increases when combining prompting configurations. Nonetheless, our findings suggest that MLLMs may currently be outperformed by humans in visual reasoning problems, even when optimized prompting configurations are used.

Our human online sample outperformed MLLMs across a suit of diverse mental rotation and figural reasoning tests. This is consistent with prior reports of below average visual reasoning performance of MLLMs (mental rotation: Hayashi & Hirata, 2026; Stogiannidis et al., 2025; figural reasoning: Cao et al., 2025; Haznitrama et al., 2026). This subpar performance appears to persist when prompting MLLMs via online interfaces instead of APIs. However, it remains unclear whether prompting via online interfaces or via APIs may yield systematic differences in MLLM visual reasoning performance (i.e., akin to systematic differences in content moderation; Lipphardt et al., 2026; for a related discussion on psychometric item generation see Marmolejo-Ramos et al., 2025).

MLLMs performed comparatively worse in mental rotation compared to figural reasoning tests. To solve mental rotation tasks, humans must perform a set of visuospatial processing steps, such as encoding features of the stimuli and comparing rotation candidates or specific stimulus areas with the original stimulus image (e.g., J. Xue et al., 2017). However, to date it is unclear at best and perhaps unlikely that MLLMs can perform visuospatial processing steps akin to humans. Therefore, MLLMs may be unable to solve such problems using the solution strategies that these measures were intended to capture.

Instead, MLLMs may use other solution strategies such as reasoning to arrive at an appropriate solution. This means that the observed MLLM performance deficits could be rooted in fundamentally different problem-solving strategies between humans and MLLMs. Humans might rotate mentally abstracted 3D representations of 2D objects to arrive at a solution for a problem that MLLMs try to solve by sequential logical reasoning. In other words, our MLLMs may have performed poorly on mental rotation tests simply because they fundamentally lack the ability to mentally rotate.

However, the single exception to this observation relates to Gemini 3’s performance on the monochromatic cube rotation test, which was comparable to human performance. One plausible explanation of this finding could be that the items consisting of cubes which comprise only three monochromatic faces may possibly be solved by mere reasoning instead of mental rotation performance. More specifically, if both stimulus and response cubes display the same three colors, but in different positions, a response cube by default cannot represent a rotated view of the stimulus cube, as long as any color is only allowed to appear once on each side of the cube. Similarly, if stimulus and response cubes show two identically colored and positioned faces, no rotation can have taken place. For the dichromatic cube test, no such rules can be reasonably deducted. Therefore, Gemini 3’s performance on the monochromatic cube test may be based on correct deduction of few logical rules (i.e., correct responses can be produced based on reasoning, rather than mental rotation) and the performance decrease on the dichromatic cube test may be rooted in the fact that reasoning is not a suitable strategy to solve these tasks. Considering that Gemini 3 more often than not outperformed ChatGPT-5 in our testing setup, ChatGPT-5 may have failed to reliably deduce or apply the logical rules, explaining why this model barely performed above chance level even on the monochromatic cube rotation test.

The MLLMs may have performed comparatively better in figural reasoning tests because they did not require to mentally rotate three-dimensional figures. Indeed, the matrix progression items are designed to be solved by deducing and applying progression rules in highly structured two-dimensional displays (i.e., 3 × 3 matrices). On the one hand, these findings may mean that progressive logical reasoning processes play more to the MLLMs’ strengths compared to visuospatial processing. On the other hand, it could be speculated that logical reasoning may be more easily translatable to verbal content, therefore becoming more accessible to MLLMs. Both ideas support the interpretation that MLLM between-domain performance differentiation is rooted in the tests’ visuospatial processing versus reasoning requirements. This pattern is also supported by the observation that MLLMs performed above chance on both single choice matrix progression tests but virtually at chance level on the visuospatially comparatively complex response assembly matrix progression test. Finally, in the latter test, the number of matrix progression rules included in an item was negatively associated with item solution probabilities. Since each rule was associated with additional but independent visual elements, this conforms to the interpretation that subpar performance is rooted in increased visuospatial complexity of items.

The possibility that incorrect MLLM visual reasoning was caused by errors during visuospatial processing rather than logical reasoning is further supported by recent empirical accounts (Cao et al., 2025; Y. Zhang et al., 2024). In fact, subpar MLLM performance in visuospatial processing has been suggested to be at least partly attributable to sequential instead of holistic processing in transformer-based MLLMs (Hayashi & Hirata, 2026). Furthermore, subtle hallucinations may occur unnoticed, as, for example, a previous study observed that MLLMs still perceived a tennis racket in an image where said racket had been edited out of a player’s hands (Villa et al., 2025). Such perceptual hallucinations in MLLMs could plausibly yield incorrect responses in visual reasoning tasks.

We note that additional, multimodal-specific error sources beyond visuospatial processing and logical reasoning factors may have affected MLLM task performance as well (Chung et al., 2026). Interestingly, error sources have been observed to shift from perceptual to reasoning factors as tasks got more complex, thus indicating that item difficulty may be systematically linked to different reasons for incorrect responses (L. Zhang et al., 2026). Future researchers may wish to contribute towards disentangling underlying mechanisms of incorrect responses in MLLM visual reasoning.

Of note, while MLLMs performed comparatively worse in mental rotation compared to figural reasoning tests, this pattern was reversed for our human online sample. This observation supports the interpretation that relative item difficulty between MLLMs and humans is dependent on cognitive domain.

Different prompting configurations that can be reasonably expected to be employed by average users tended to improve MLLM performance compared to the model-specific default prompting specifications in the online interfaces. While self-consistency, context (item-wise prompting), and CoT configurations yielded mean test score improvements in both MLLMs, segmented uploads and English (compared to German) instructions yielded improved performance only for ChatGPT-5, but reduced performance for Gemini 3. Improved performance via prompting configurations is in line with previous studies which observed improved reasoning using certain prompting configurations (e.g., segmented inputs: Li et al., 2025; self-consistency: Wang et al., 2023; CoT: Wei et al., 2022; but see also Yang et al., 2025, where prompting configurations such as self-consistency and CoT did not improve performance). Importantly, the combination of best-performing prompting configurations yielded the largest mean score improvements in both MLLMs. Consequently, our study indicates that optimal prompting strategies comprise a combination of multiple single prompting configurations.

We prompted MLLMs via online interfaces, ensuring relatively naturalistic testing conditions considering expected average users. This may represent an important step toward evaluating MLLM capabilities available to average users via web-based testing instead of API-based testing (Lipphardt et al., 2026; Park et al., 2025). This approach, combined with our systematic assessment of prompting configurations, increases the ecological validity of our findings by ensuring their informative value when considering the role of MLLMs for current average visual reasoning use cases. These prominently include unproctored online assessments, but also daily tasks involving visual reasoning where users might look for assistance from MLLMs.

In a practical context, this means that MLLM-assisted responses appear currently relatively unproblematic for unproctored visual reasoning assessments due to their suboptimal performance. However, given the recent rapid technological advances in the field, this might change with future MLLM versions (though increasing the volume of training data alone might quickly yield diminishing returns; W. Zhang et al., 2025), and continuous monitoring seems warranted.

### Limitations

Some limitations of the present study need to be acknowledged. First, the use of an online convenience sample limits the generalizability of our findings, because it seems plausible that our self-selected participants performed above the population average, owing to typical limitations of online samples (Andrade, 2020). Of note, the presently encountered ceiling effects in multiple tests raise the possibility that our human online sample might have outperformed MLLMs by even greater margins in harder tests. Furthermore, manual prompting in MLLMs did not allow for re-running most prompting configurations, thus possibly reducing precision of performance estimations. However, performance in the self-consistency configuration (ten runs) showed that our results remained reasonably stable between different runs (mean raw score changes compared to default prompting of 0.83 and 1.00 points for ChatGPT-5 and Gemini 3, respectively), thus largely alleviating concerns about between-runs response behavior variability. Finally, it is possible that matrix progression tests were more abundant in MLLM training data compared to mental rotation tests, thereby providing an alternative explanation for comparatively better MLLM figural reasoning performance in our data. However, the abundance of online accessible mental rotation and figural reasoning tests limits the explanatory value of this idea.

## 5. Conclusions

In all, a human online sample outperformed modern, widely used MLLMs in mental rotation and figural reasoning tasks across different tests and prompting configurations in naturalistic settings (identical instructions, testing via online interfaces instead of APIs). It is possible that the surprisingly poor MLLM performance may be linked to suboptimal MLLM visuospatial processing performance rather than logical reasoning, although further empirical tests of this idea are needed. Without a doubt, it can be expected that MLLM performance will continue to improve in the future, thus warranting continuous monitoring of the rapidly evolving effects of MLLM assistance for common visuospatial and reasoning tasks.

## Figures and Tables

**Table 1:** Characteristics of human participants per test.

Test	*N*	*n* f/m/d	Mean Age ^a^ (*SD*)	Education				
				1	2	3	4	5
R-Cube-SR-1	187	114/68/5	31.97 (13.99)	0	9	10	99	69
R-Cube-SR-2	153	96/53/4	31.19 (13.39)	0	6	7	83	57
3D-FR	175	104/65/6	29.88 (12.17)	1	11	9	90	64
HMT	121	72/45/4	30.94 (12.96)	1	6	6	62	46
MaRs	202	127/68/7	31.31 (13.37)	0	10	9	103	80
OMIB	91	59/31/1	30.03 (12.39)	0	5	4	54	28

^a^ Mean age based on 410 total participants. Note. R-Cube-SR-1/2 = cube rotation test with monochromatic faces and dichromatic faces, respectively; 3D-FR = figure rotation test; HMT = black-and-white matrix progression test; MaRs = colored matrix progression test; OMIB = response assembly matrix progression test; f = female, m = male, d = diverse; Education = highest educational attainment: 1 (no school degree or special education, <9 years of education), 2 (mandatory schooling finished, ~9–10 years of education), 3 (vocational school degree, ~10–12 years of education), 4 (high school finished, ~12–13 years of education), 5 (university degree, >13 years of education).

**Table 2:** Test Scores of MLLMs and humans on five tests of non-verbal reasoning.

		MLLMs and Prompting Configurations								Human Online Sample
	Default	Self-Cons.	Segmentation	Context	CoT	English	Comb.	Max. Score	Comb/idk (Count “Idk”)	Mean (95% CI)
		ChatGPT-5								
R-Cube-SR-1	14	12.5	9	20	16	12	11	24	14 (0)	23.15 (22.86; 23.44)
R-Cube-SR-2	7	9.5	8	10	12	9	12	24	11 (3)	21.93 (21.56; 22.30)
3D-FR	13	12.5	12	14	12	15	19	24	17 (0)	22.62 (22.30; 22.94)
HMT	2	5.5	9	3	4	4	13	20	11 (0)	12.31 (11.50; 13.11)
MaRs	12	12	14	13	10	10	15	20	17 (0)	18.28 (18.04; 18.51)
OMIB	4	5	5	6	4	6	5	28	6 (15)	19.64 (18.03; 21.24)
		Gemini 3 Thinking								
R-Cube-SR-1	24	24	23	24	24	22	24	24	24 (0)	23.15 (22.86; 23.44)
R-Cube-SR-2	11	13	14	12	10	11	18	24	13 (0)	21.93 (21.56; 22.30)
3D-FR	14	13.5	12	15	18	17	17	24	16 (0)	22.62 (22.30; 22.94)
HMT	12	12	7	12	16	10	12	20	10 (0)	12.31 (11.50; 13.11)
MaRs	15	18.5	11	18	16	12	18	20	18 (0)	18.28 (18.04; 18.51)
OMIB	5	6	4	4	6	5	6	28	6 (0)	19.64 (18.03; 21.24)

Note. R-Cube-SR-1/2 = cube rotation test with monochromatic faces and dichromatic faces, respectively; 3D-FR = figure rotation test; HMT = black-and-white matrix progression test; MaRs = colored matrix progression test; OMIB = response assembly matrix progression test. Self-cons. = self-consistency, CoT = Chain-of-Thought, Comb. = combination of prompting configurations, Comb/idk = combination of prompting configurations with the additional response option “I don’t know.”. One run per prompting configuration, test, and MLLM, excepting self-consistency (10 runs). Partial points in the self-consistency prompting configuration resulted from ties during response majority voting.

## Data Availability

Test data from human participants, all MLLM responses, the analysis script, and the Supplementary Materials are available at https://osf.io/f4nqb/files/osfstorage (accessed on 4 September 2026).

## Supplementary Materials

Test data from human participants, all MLLM responses, the analysis script, and the Supplementary Materials can be downloaded at https://osf.io/f4nqb/files/osfstorage (accessed on 4 September 2026). The following Supplementary Materials are available: analysis.R: the full analysis script required to reproduce the findings reported in this study; humanData.xlsx: the anonymized test data from our human online sample; Masterfile_solutions.xlsx: details for constructing our items based on the original open-access stimuli files as well as keys to scoring these items; MLLM_scoring.xlsx: the test data from MLLMs with detailed timestamps; preregDeviations.docx: details on deviations from the preregistration; supplementaryMaterials.docx: additional details on sample exclusions, test licensing and modifications from original open-access test materials, used R packages, instructions to humans and MLLMs including verbatim prompts, quantitative results on matrix progression rule tests, and boxplot/scatterplot visualizations of results.

## Abbreviations

The following abbreviations are used in this manuscript:

MLLM	Multimodal large language model
R-Cube-SR	Cube mental rotation test
3D-FR	Figure mental rotation test
HMT	Black-and-white matrix progression test
MaRs	Colored matrix progression test
OMIB	Response assembly matrix progression test
CoT	Chain-of-Thought
